# Specific association of TBK1 with the trans-Golgi network following STING stimulation

**DOI:** 10.1247/csf.21080

**Published:** 2022-02-05

**Authors:** Haruka Kemmoku, Yoshihiko Kuchitsu, Kojiro Mukai, Tomohiko Taguchi

**Affiliations:** 1 Laboratory of Organelle Pathophysiology, Department of Integrative Life Sciences, Graduate School of Life Sciences, Tohoku University, Sendai, Japan

**Keywords:** the Golgi, membrane traffic, innate immunity, STING

## Abstract

Stimulator of interferon genes (STING) is essential for the type I interferon response induced by microbial DNA or self-DNA leaked from mitochondria/nuclei. In response to the emergence of such DNAs in the cytosol, STING relocates from the endoplasmic reticulum (ER) to the Golgi, and activates TANK-binding kinase 1 (TBK1), a cytosolic kinase essential for the activation of STING-dependent downstream signalling. To understand at which subcellular compartments TBK1 becomes associated with STING, we generated cells stably expressing fluorescent protein-tagged STING (mNeonGreen-STING) and TBK1 (TBK1-mScarletI). We found that after STING stimulation, TBK1 became associated with the trans-Golgi network (TGN), not the other parts of the Golgi. STING variants that constitutively induce the type I interferon response have been identified in patients with autoinflammatory diseases named “STING-associated vasculopathy with onset in infancy (SAVI)”. Even in cells expressing these constitutively active STING variants, TBK1 was found to be associated with TGN, not the other parts of the Golgi. These results suggest that TGN acts as a specific platform where STING associates with and activates TBK1.

## Introduction

The detection of microbial pathogens with nucleic acid sensors is one of the central strategies of innate immunity (Palm and Medzhitov, 2009; Takeuchi and Akira, 2010). Cyclic GMP-AMP synthase (cGAS) is a sensor for double-stranded DNA in the cytosol (Sun *et al.*, 2013). It synthesizes cyclic GMP-AMP (cGAMP) with ATP and GTP (Wu *et al.*, 2013), which stimulates the induction of type I interferons and proinflammatory cytokines through the cGAMP sensor STING (Ishikawa and Barber, 2008) [also known as MITA (Zhong *et al.*, 2008), ERIS (Sun *et al.*, 2009), MPYS (Jin *et al.*, 2008), or TMEM173]. STING is postulated to act as a protein scaffold to activate the downstream protein kinase TBK1 (Ishikawa *et al.*, 2009; Saitoh *et al.*, 2009; Tanaka and Chen, 2012; Zhang *et al.*, 2019). Activated TBK1 phosphorylates and thus activates IRF3, the essential transcription factor that induces the synthesis of type I interferons (Fitzgerald *et al.*, 2003). During this process, TBK1 also phosphorylates STING, generating the IRF3-docking site on STING (Liu *et al.*, 2015).

STING is an ER-localized transmembrane protein (Ishikawa and Barber, 2008). After its binding to cGAMP, STING exits the ER with COP-II vesicles. We and others have shown that the COP-II-mediated translocation of STING from the ER was required to activate the downstream signalling pathway (Dobbs *et al.*, 2015; Mukai *et al.*, 2016; Ogawa *et al.*, 2018; Gui *et al.*, 2019; Sun *et al.*, 2018; Ran *et al.*, 2019). In two autoinflammatory monogenic diseases, STING-associated vasculopathy with onset in infancy (SAVI) (Liu *et al.*, 2014) and the COPA syndrome (Watkin *et al.*, 2015), STING was constitutively active without DNA stimulation and localized not to the ER but to the perinuclear compartments including the Golgi (Jeremiah *et al.*, 2014; Mukai *et al.*, 2016; Ogawa *et al.*, 2018; Lepelley *et al.*, 2020; Deng *et al.*, 2020; Mukai *et al.*, 2021; Taguchi *et al.*, 2021). Palmitoylation of STING at the Golgi was essential to activate the downstream signalling pathway (Mukai *et al.*, 2016; Hansen *et al.*, 2018; Haag *et al.*, 2018; Jia *et al.*, 2020). Phosphorylated TBK1, the active form of TBK1, was exclusively localized to a subdomain within TGN (Mukai *et al.*, 2016). Together, these results establish that the Golgi is an organelle at which STING activates TBK1 for triggering the innate immune response (Mukai *et al.*, 2016; Taguchi and Mukai, 2019). However, it remains unknown how the very initial step of TBK1 activation, *i.e*., the recruitment of TBK1 to STING, occurs during the STING relocalization from the ER to the Golgi.

In the present study, we develop a cell system by which STING and TBK1 are simultaneously monitored. We demonstrate that TBK1 association with STING occurs exclusively at TGN, not the other parts of the Golgi.

## Results

### Generation and validation of cells with fluorescent protein-tagged TBK1 and STING

To monitor the dynamics of TBK1 and STING, we tagged TBK1 and STING with mScarletI and mNeonGreen, respectively. Mouse embryonic fibroblasts (MEFs) were chosen since the cGAS/STING pathway is active in these cells (Ishikawa and Barber, 2008). We prepared STING/TBK1 double knockout MEFs (ST-DKO MEFs) from STING knockout MEFs using the CRISPR-Cas9 technology to eliminate the contribution of endogenous STING and TBK1. ST-DKO MEFs were then reconstituted with *C*-terminally mScarletI-tagged TBK1 and *N*-terminally mNeonGreen-tagged STING.

**Fig. 1:**
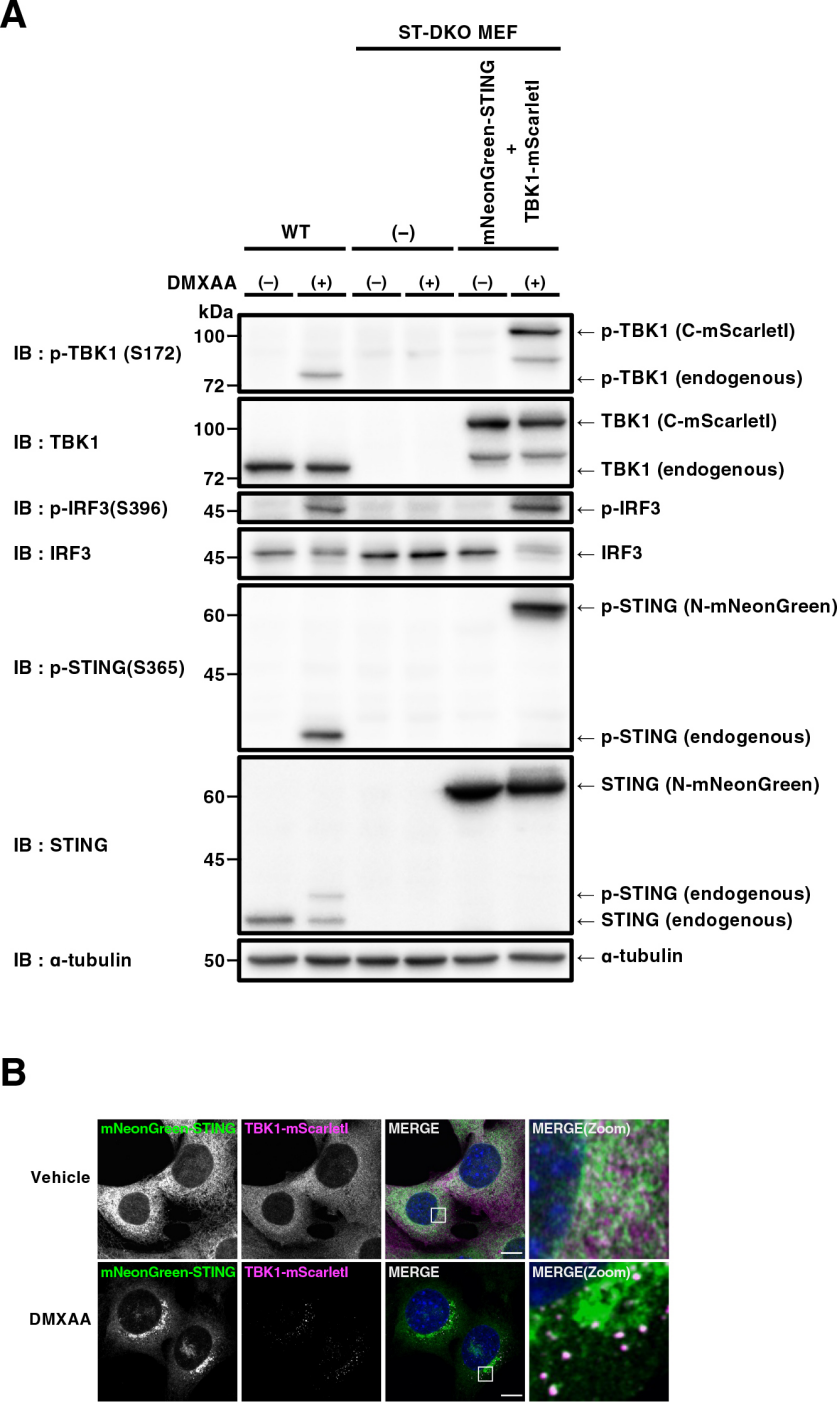
Generation of mNeonGreen-STING- and TBK1-mScarletI-reconstituted MEFs (A) STING/TBK1 double-knockout MEFs (ST-DKO MEFs) were generated from STING-knockout MEFs using the CRISPR-Cas9 technology. ST-DKO MEFs were reconstituted with mNeonGreen-STING and TBK1-mScarletI. The reconstituted ST-DKO MEFs were stimulated with DMXAA (25 μg/mL) for 60 min. Cell lysates were prepared and analyzed by western blot. (B) mNeonGreen-STING- and TBK1-mScarletI-expressing ST-DKO MEFs were stimulated with DMXAA (25 μg/mL) for 60 min, fixed, permeabilized, and stained with DAPI (blue). Scale bar, 10 μm.

We first validated if fluorescent protein-tagged STING and TBK1 were functional. Cells were stimulated with DMXAA, a membrane-permeable mouse-specific STING agonist (Prantner *et al.*, 2012). As expected, 60 min after DMXAA stimulation, we observed phosphorylated TBK1 at Ser172 (p-TBK1), phosphorylated STING at Ser365 (p-STING), and phosphorylated IRF3 at Ser396 (p-IRF3) in wild-type (WT) MEFs, but not in ST-DKO MEFs (Fig. 1A). In ST-DKO MEFs reconstituted with mNeonGreen-STING and TBK1-mScarletI, phosphorylated TBK1-mScarletI, phosphorylated mNeonGreen-STING, and p-IRF3 emerged after DMXAA stimulation (Fig. 1A), showing that reconstituted two proteins were functional. We observed long and short TBK1 (Fig. 1A). The long TBK1, not the short TBK1, contained mScarletI (Fig. S1A), indicating that (i) the long one corresponded to TBK1-mScarletI and (ii) the short one was generated by the truncation of mScarletI tag of TBK1-mScarletI.

As with endogenous STING (Konno *et al.*, 2013) and TBK1 (Ishikawa *et al.*, 2009; Saitoh *et al.*, 2009; Wang *et al.*, 2014; Ogawa *et al.*, 2018), mNeonGreen-STING and TBK1-mScarletI showed typical ER and cytosolic localization in unstimulated cells, respectively (Fig. 1B). After DMXAA stimulation, these two proteins drastically changed their subcellular localizations. mNeonGreen-STING relocated to the perinuclear compartments, as endogenous STING (Konno *et al.*, 2013). TBK1-mScarletI localized at multiple puncta which were positive with mNeonGreen-STING (Fig. 1B). The immunostaining of TBK1 also showed multiple puncta which were positive with mScarletI (Fig. S1B).

### Exclusive association of TBK1 with TGN following STING stimulation

We sought to examine at which subcellular compartments TBK1 became associated with STING. The Golgi is a polarized organelle that has distinct functional domains, such as the cis-Golgi network (CGN) and TGN. Treatment of cells with a microtubule-depolymerizing agent nocodazole resulted in dispersed Golgi stacks “mini-Golgi” in the cytoplasm, and this fragmentation facilitated the analysis of *cis*-to-*trans* polarity of the Golgi (Shima *et al.*, 1997).

**Fig. 2:**
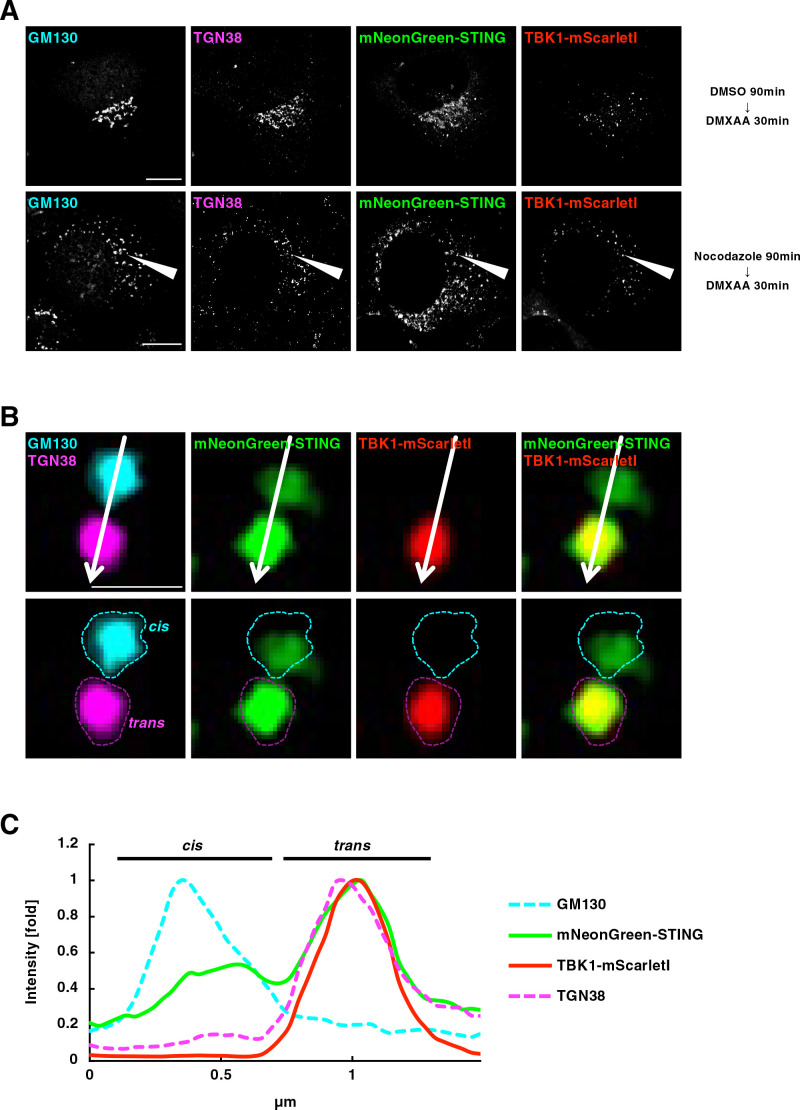
TBK1 association with TGN in mini-Golgi (A) mNeonGreen-STING- and TBK1-mScarletI-reconstituted ST-DKO MEFs were treated with nocodazole (2.5 μM) for 90 min, followed by stimulation with DMXAA (25 μg/mL) for 30 min. Cells were fixed, permeabilized, and stained for GM130 (a CGN protein, cyan) and TGN38 (a TGN protein, magenta). Scale bars, 10 μm. (B) One mini-Golgi indicated by arrowhead in (A) was magnified. The *cis*- and *trans*-regions of the mini-Golgi were outlined in the images at the bottom row. Scale bar, 1 μm. (C) Fluorescence intensity profile along the arrows in (B) is shown. See also Fig. S1.

We exploited this strategy to scrutinize the membrane relocalization of TBK1 after STING stimulation. We treated cells with nocodazole for 90 min and then with DMXAA for 30 min. Cells were then fixed and stained for GM130 (a CGN protein) and TGN38 (a TGN protein). As expected, both GM130 and TGN38 were scattered throughout the cytoplasm (Fig. 2A). As a representative example, one mini-Golgi indicated by arrowheads was chosen (Fig. 2A) and analyzed (Fig. 2B and 2C). Within the mini-Golgi, mNeonGreen-STING localized at both CGN and TGN, whereas TBK1-mScarletI was confined to TGN. Analysis of multiple mini-Golgis (n=30, 5 cells) also showed the exclusive confinement of TBK1-mScarletI to sub-domains within TGN (Fig. S2).

### Exclusive association of TBK1 with TGN in cells expressing the SAVI variants

The STING variants (V147L, N154S, V155 M, C206Y, R281Q, or R284G) that constitutively induced type I interferon response were identified in the SAVI patients (Liu *et al.*, 2014; Jeremiah *et al.*, 2014; Melki *et al.*, 2017). Even in the absence of STING ligand, the SAVI variants exited the ER and relocated to the Golgi at which the SAVI variants activated TBK1 (Jeremiah *et al.*, 2014; Mukai *et al.*, 2016; Ogawa *et al.*, 2018). Besides the Golgi, the SAVI variants localize at recycling endosomes (Fig. S3).

**Fig. 3:**
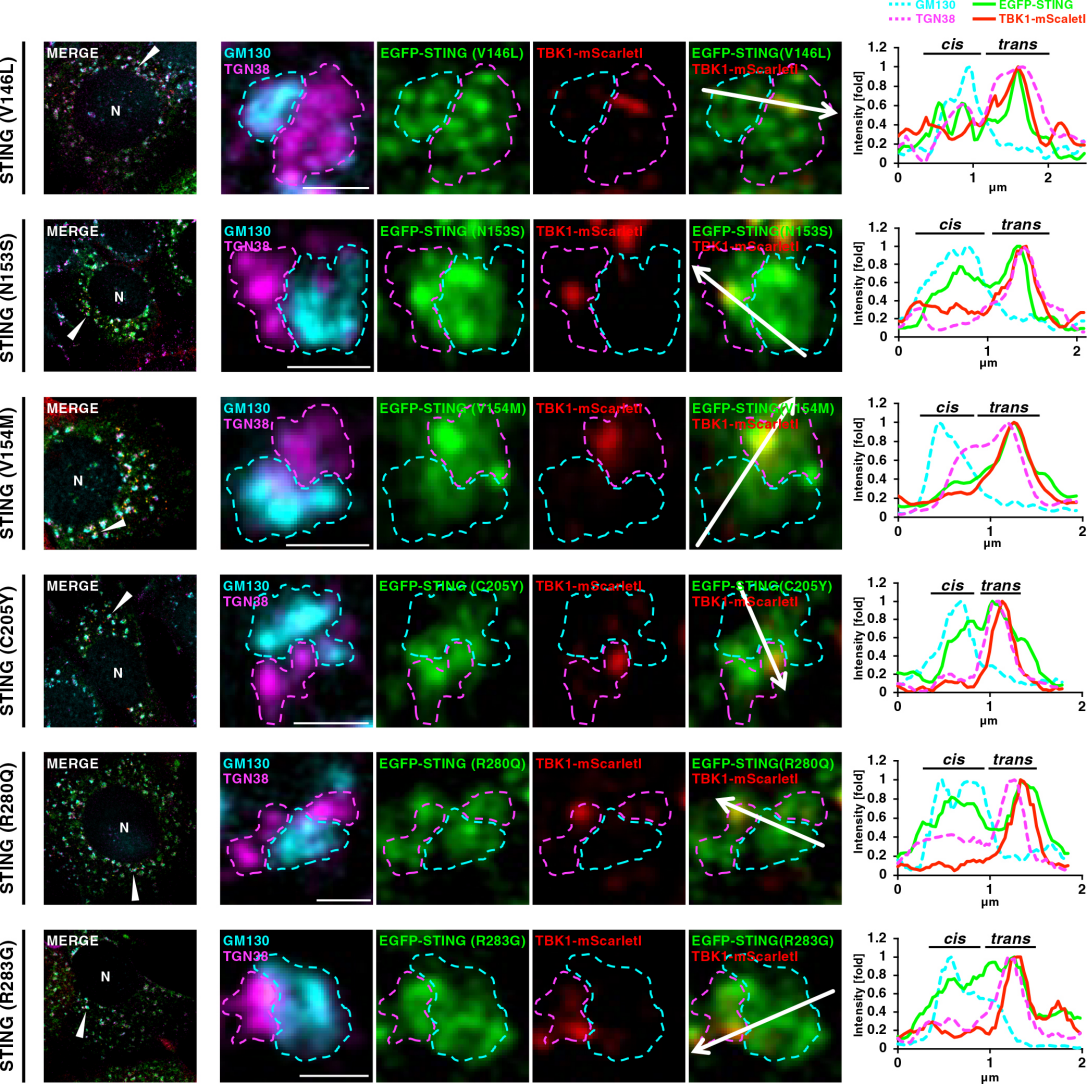
TBK1 association with TGN in mini-Golgi in cells expressing the SAVI-variants EGFP-STING (SAVI)- and TBK1-mScarletI-reconstituted ST-DKO MEFs were treated with BFA (3 μg/mL) for 3 h and further incubated with BFA-free growth medium for 45 min, and then fixed. Nocodazole was added to the culture medium 75 min before fixation. Fixed cells were permeabilized, and stained for GM130 (a CGN protein, cyan) and TGN38 (a TGN protein, magenta). Scale bars, 1 μm. Each mini-Golgi indicated by arrowhead in the left column was magnified. The *cis*- and *trans*-regions of the mini-Golgi at the magnified images were outlined. Fluorescence intensity profiles along the arrows are shown. See also Fig. S2.

We sought to examine if the SAVI variants also became associated with TBK1 specifically at TGN. To address this issue, it was ideal to analyze the cells in which the membrane traffic of the SAVI variants from the ER was synchronized. We exploited brefeldin A (BFA), a fungal macrocyclic lactone that blocks ER-to-Golgi traffic (Lippincott-Schwartz *et al.*, 1990). Cells expressing the six different murine equivalent SAVI variants (V146L, N153S, V154M, C205Y, R280Q, or R283G) tagged with EGFP, were first treated with BFA for 3 hours. Cells were then released from the BFA block, incubated with nocodazole for another 45 minutes, fixed, and stained for GM130 and TGN38. As a representative example, one mini-Golgi was chosen from cells expressing individual SAVI variants, and analyzed (Fig. 3). We found that TBK1-mScarletI localized exclusively to a sub-domain within TGN, whereas the SAVI variants localized at both CGN and TGN (Fig. 3 and Fig. S4). These data suggested that the disease-causative STING variants, besides WT STING, became associated with TBK1 at TGN, not the other parts of the Golgi.

### Association of TBK1 with TGN in cells not treated with nocodazole

We sought to examine if TBK1 became associated with TGN in cells that were not treated with nocodazole. To this end, we used Airyscan super-resolution microscopy. Airyscan is a 32-channel gallium arsenide phosphide photomultiplier tube (GaAsP-PMT) area detector that collects a pinhole-plane image at every scan position, providing lateral resolution to even 120 nm for two-dimensional imaging (Huff *et al.*, 2017). To visualize CGN or TGN in live cells, *N*-terminally mScarletI-tagged GM130 or Rab6 (a TGN protein) was used. With Airyscan super-resolution microscopy, CGN and TGN could be resolved, although not completely, as indicated by different localization profiles between mScarletI-GM130 (CGN) and TGN38 (TGN) or between GM130 (CGN) and mScarletI-Rab6 (TGN) (Fig. S5).

**Fig. 4:**
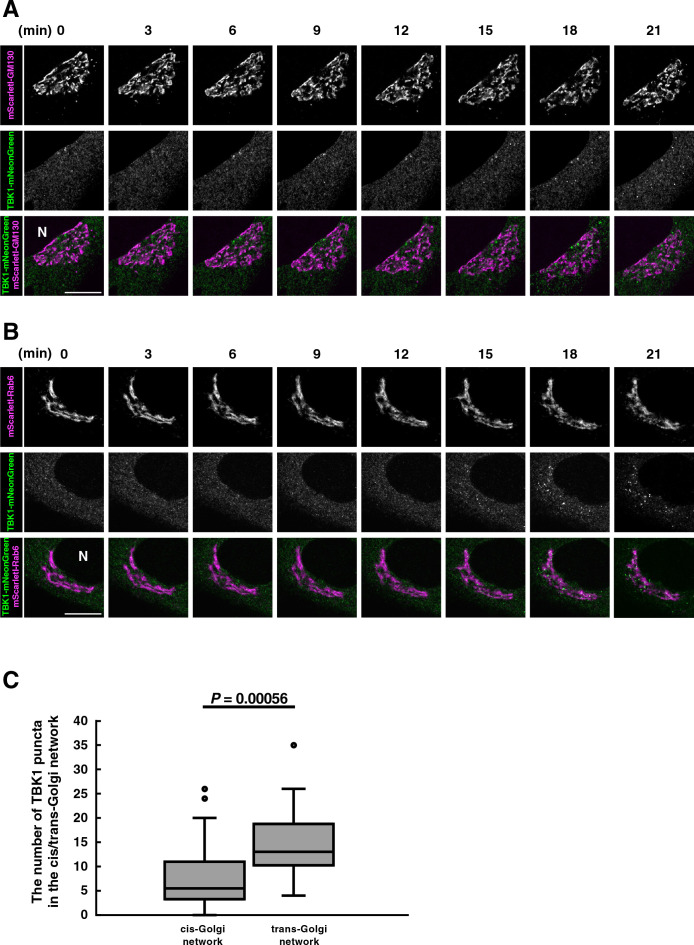
TBK1 translocates from the cytosol to TGN in living cells (A, B) TBK1-knockout MEFs were reconstituted with TBK1-mNeonGreen using retrovirus. mScarletI-GM130 (A) or mScarletI-Rab6 (B) was stably expressed in the reconstituted TBK1-knockout MEFs. Cells were then stimulated with DMXAA (25 μg/mL). Live-cell images were taken at 30-sec intervals by fluorescence microscopy. Selected frames from the movie are shown. N, the nucleus. Scale bar, 10 μm. (C) The number of TBK1 puncta inside CGN (mScarletI-GM130) or TGN (mScarletI-Rab6) 20-35 min after stimulation was measured. Data are presented in box-and-whisker plot with the minimum, maximum, sample median, and first vs. third quartiles (CGN n=23 cells; TGN n=27 cells). Statistical significance was determined with two-tailed Student’s *t*-test. See also Fig. S4.

Cells were stimulated with DMXAA and analyzed at indicated times by Airyscan super-resolution microscopy (Fig. 4). TBK1-mNeonGreen started to form multiple perinuclear puncta 18 min after DMXAA stimulation (Fig. 4A and 4B). The individual puncta were examined if they were within CGN or TGN (Fig. S6). We found that the number of TBK1 puncta on TGN was significantly larger than that on CGN (Fig. 4C). These results suggested that TBK1 became associated with STING at TGN, not the other parts of the Golgi in live cells.

### Kinase activity of TBK1 is dispensable for its membrane association

**Fig. 5:**
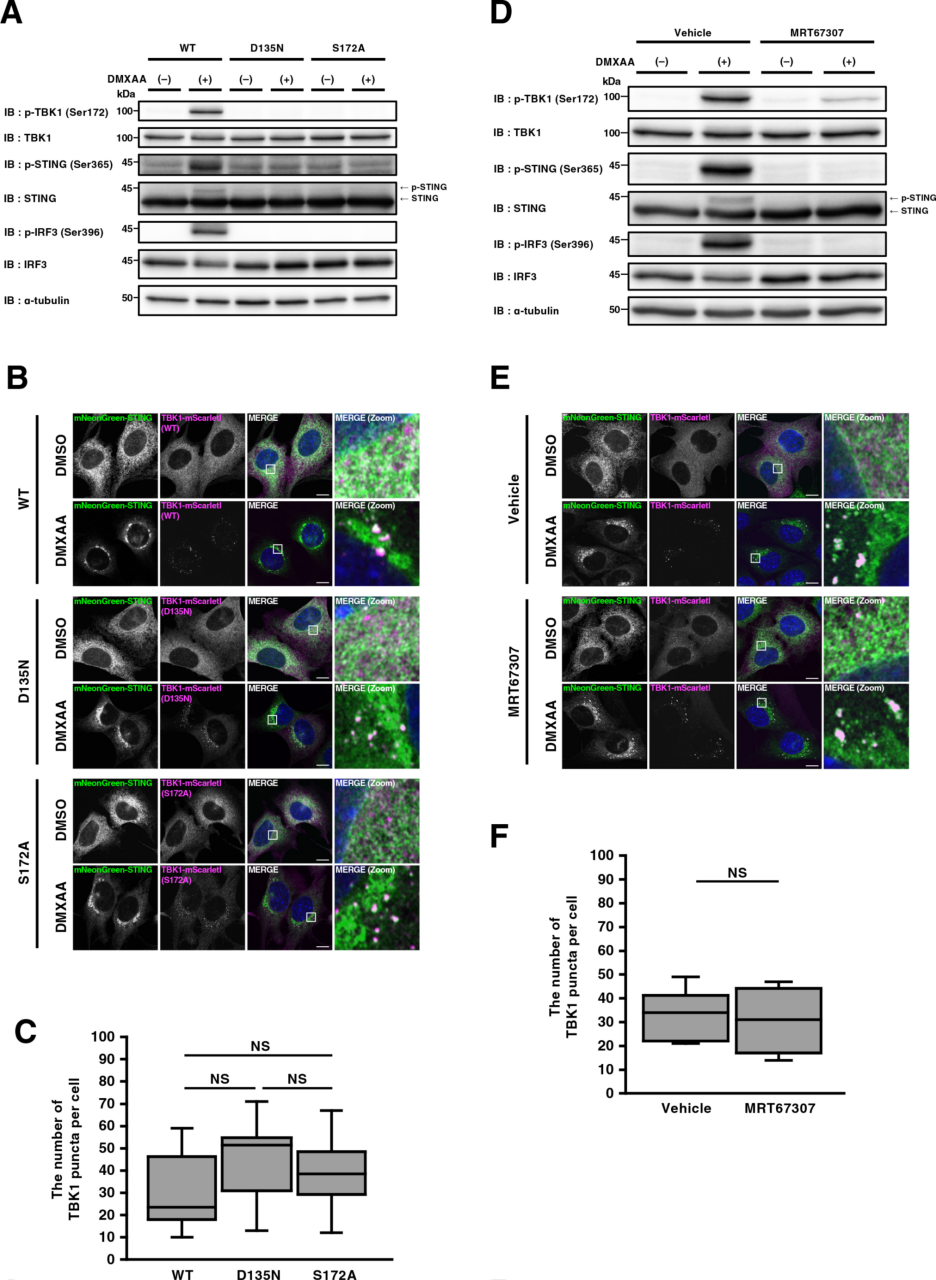
Kinase activity of TBK1 is dispensable for its membrane association (A) ST-DKO MEFs reconstituted with FLAG-STING and TBK1 (WT, D135N, or S172A)-mScarletI were stimulated with DMXAA (25 μg/mL) for 60 min. Cell lysates were prepared and analyzed by western blot. (B) ST-DKO MEFs reconstituted with mNeonGreen-STING and TBK1(WT, D135N, or S172A)-mScarletI were stimulated with DMXAA (25 μg/mL) for 60 min, fixed, permeabilized, and stained with DAPI (blue). Scale bar, 10 μm. (C) The number of TBK1 puncta after DNXAA stimulation in (B) was quantified. Data are from 10 cells. Statistical significance was determined with Tukey-Kramer’s test. (D) ST-DKO MEFs reconstituted with FLAG-STING and TBK1-mScarletI were treated with vehicle or MRT67307 (10 μM) for 2 h, and then stimulated with DMXAA (25 μg/mL) for 60 min. Cell lysates were prepared and analyzed by western blot. (E) ST-DKO MEFs reconstituted with mNeonGreen-STING and TBK1-mScarletI were treated with vehicle or MRT67307 (10 μM) for 2 h, and then stimulated with DMXAA (25 μg/mL) for 60 min. Cells were fixed, permeabilized, and stained with DAPI (blue). Scale bar, 10 μm. (F) The number of TBK1 puncta after DNXAA stimulation in (E) was quantified. Data are from 10 cells. Statistical significance was determined with two-tailed Student’s *t*-test.

We sought to determine if the kinase activity of TBK1 is involved in its membrane association at TGN. Kinase-dead variant (D135N) or autophosphorylation-deficient variant (S172A) of TBK1 (Ye *et al.*, 2019), and FLAG-STING were stably expressed in ST-DKO MEFs. After stimulation with DMXAA for 60 min, p-TBK1, p-STING, and p-IRF3 emerged in MEFs expressing WT-TBK1, but not in MEFs expressing TBK1 (D135N) or TBK1 (S172A) (Fig. 5A), in accordance with the findings that TBK1 is a critical kinase for STING (Ser365) and IRF3 (Ser396) (Liu *et al.*, 2015). We then examined the association of these TBK1 variants to the Golgi. After DMXAA stimulation, TBK1 (D135N) and TBK1 (S172A), as WT-TBK1, relocated from the cytosol to multiple puncta (Fig. 5B). At these puncta, these TBK1 variants co-localized with STING. The number of the TBK1 puncta did not differ between WT-TBK1, TBK1 (D135N), and TBK1 (S172A) (Fig. 5C). These results suggested that kinase activity of TBK1 was dispensable for its association with TGN.

MRT67307, a TBK1-specific inhibitor (Clark *et al.*, 2011), suppressed the emergence of p-TBK1, p-STING, and p-IRF3 (Fig. 5D). Under this experimental condition with MRT67307, TBK1-mScarletI relocated from the cytosol to multiple puncta (Fig. 5E). The number of the TBK1 puncta did not differ between vehicle-treated and MRT67307-treated cells (Fig. 5F). These results corroborated the notion that kinase activity of TBK1 was dispensable for its association with TGN.

## Discussion

STING is an ER-localized protein. In response to the emergence of cytosolic DNA, STING triggers the innate immune response against DNA pathogens through the activation of TBK1 (kinase) and IRF3 (transcription factor). Intriguingly, a number of recent studies have suggested that the relocation of STING from the ER to the Golgi was required to activate TBK1/IRF3. For example, autophosphorylation of TBK1 was suppressed in cells treated with BFA (Konno *et al.*, 2013; Mukai *et al.*, 2016), or cells depleted of the COP-II components (Ogawa *et al.*, 2018; Gui *et al.*, 2019; Sun *et al.*, 2018). Phosphorylation of IRF3 was drastically suppressed in the conditions where STING palmitoylation at the Golgi was inhibited (Mukai *et al.*, 2016). Phosphorylated TBK1 was exclusively localized to a subdomain within TGN (Mukai *et al.*, 2016). Despite these advances, how the very initial step of TBK1 activation, *i.e*., the recruitment of TBK1 to STING, proceeds remains unknown.

In the present study, to address at which subcellular compartments TBK1 becomes associated with STING, we developed a cell system by which STING and TBK1 were simultaneously monitored. We found that the membrane localization of TBK1 was only observed at TGN, not the other parts of the Golgi, despite the STING localization throughout the entire Golgi (Fig. 2 and Fig. 4). Thus, only STING at TGN appeared capable of being associated with TBK1. The disease-causative STING variants, which activate TBK1/IRF3 without cGAMP, were also capable of being associated with TBK1, only when the STING variants were at TGN (Fig. 3). These results demonstrate that TGN acts as a specific platform where STING associates with and activates TBK1.

STING is postulated to become clustered at TGN (Mukai *et al.*, 2016; Haag *et al.*, 2018). The affinity of palmitoylated proteins to the TGN raft lipids consisting of sphingomyelin and cholesterol (Levental *et al.*, 2010; Duran *et al.*, 2012) may underlie the STING polymerization at TGN (Takahashi *et al.*, 2021). Sulfated glycosaminoglycan may also drive the STING clusterization at TGN (Fang *et al.*, 2021). The clustering of STING at TGN will increase the local concentration of STING on the TGN membrane, which may facilitate the recruitment of and/or stable association with TBK1 at TGN. The multiple TBK1 proteins on the single STING cluster may phosphorylate efficiently each other, leading to full activation of TBK1. Intriguingly, the kinase activity of TBK1 was dispensable for the formation of TBK1 puncta at TGN (Fig. 5). These results suggested that the clustering of STING proceeds independently of the kinase reaction of TBK1.

Recent studies showed that a conserved PLPLR[T/S]D motif within the *C*-terminal tail of STING mediates the activation of TBK1 (Zhao *et al.*, 2019). It remains to be elucidated how the *C*-terminal tail of STING can associate with TBK1 when STING localizes at TGN, not the other subcellular compartments including the rest of the Golgi and the ER.

## Methods

### Antibodies

Antibodies used in this study were as follows: rabbit anti-phospho-STING (D8F4W, dilution 1:1,000), rabbit anti-phospho-TBK1 (D52C2, dilution 1:1,000), rabbit anti-IRF3 (D83B9, dilution 1:1000), rabbit anti-phospho-IRF3 (4D4G, dilution 1:1,000), rabbit anti-Rab11 (D4F5, 1:100) (Cell Signaling Technology); rabbit anti-TBK1 (ab40676, dilution 1:1,000 for western blot and dilution 1:200 for immunofluorescence) (Abcam); mouse anti-α-tubulin (DM1A, dilution 1:1,000) (Sigma-Aldrich); Goat Anti-Rabbit IgG (H+L) Mouse/Human ads-HRP (4050-05, dilution 1:10,000) and Goat Anti-Mouse IgG (H+L) Human ads-HRP (1031-05, dilution 1:10,000) (Southern Biotech); sheep anti-TGN38 (AHP499G, dilution 1:200) (Bio-Rad); mouse anti-GM130 (610823, dilution 1:2,000) (BD Biosciences); rabbit anti-mCherry (PA5-34974, dilution 1:1,000), Alexa 647-conjugated secondary antibodies (A21448, dilution 1:1,000), Alexa 647-conjugated secondary antibodies (A31573, dilution 1:1,000) (Thermo Fisher Scientific); DyLight 405-conjugated secondary antibodies (715-475-151, dilution 1:200) (Jackson ImmunoResearch). The antibody against STING was generated by immunizing rabbit with recombinant glutathione S-transferase (GST)-hSTING-C (amino acids 173–379) produced in *E. coli*.

### Reagents

The following reagents were purchased from the manufacturers as noted: DMXAA (14617, Cayman); Nocodazole (13857, Cayman); Brefeldin A (11861, Cayman); MRT67307 (19916, Cayman).

### PCR cloning

*N*-terminal mNeonGreen- or EGFP -tagged mouse STING (NM_028261) was introduced into pMXs-IPuro. *C*-terminal mScarletI- or mNeonGreen-tagged human TBK1 (NM_013254) was introduced into pMXs-IBla. The STING variants (V146L, N153S, V154M, C205Y, R280Q, or R283G) were generated by site-directed mutagenesis. Mouse GM130 and Rab6 were amplified by PCR with complementary DNA derived from C57BL/6J mouse thymus. The product encoding mouse GM130 was introduced into pMXs-IPuro-mScarletI to generate *N*-terminal mScarletI-tagged GM130. The product encoding mouse Rab6 was introduced into pMXs-IHyg-mScarletI to generate *N*-terminal mScarletI-tagged Rab6.

### Cell culture

MEFs were obtained from embryos of WT or *Sting^–/–^* mice at E13.5 and immortalized with SV40 Large T antigen. MEFs were cultured in DMEM supplemented with 10% fetal bovine serum (FBS) and penicillin/streptomycin/glutamine (PSG) in a 5% CO_2_ incubator. MEFs that stably express tagged proteins were established using retrovirus. Plat-E cells were transfected with pMXs vectors, and the medium that contains the retrovirus was collected. MEFs were incubated with the medium and then selected with puromycin (2 μg/mL), blasticidin (5 μg/mL), or hygromycin (400 μg/mL) for several days.

### Generation of STING- and TBK1-double knockout cells by CRISPR-Cas9

Single-guide RNA (sgRNA) was designed to target mouse TBK1 genomic loci. The sgRNA (sense: 5'-caccgGAGGAGCCGTCCAATGCGTA-3', antisense: 5'-aaacTACGCATTGGACGGCTCCTCc) was cloned into pSpCas9 (BB)-2A-mCherry created by PX459 V2.0 (Addgene #62988). This plasmid was then transfected into immortalized STING-knockout MEFs (Mukai *et al.*, 2016) with PEI MAX (24765-1, Polyscience). Twenty-four hours after transfection, mCherry expressing cells were sorted by flow cytometry (SH800, SONY). Single colonies were then isolated by limited dilution cloning and the expression of TBK1 in each clone was analyzed by western blot.

### Immunocytochemistry

Cells were fixed with 4% paraformaldehyde (PFA) in PBS at room temperature for 15 min, permeabilized with 0.1% Triton X-100 in PBS at room temperature for 5 min. After blocking with 3% BSA in PBS, cells were incubated with primary antibodies. After washing with PBS three times, cells were then incubated with the secondary antibody at room temperature for 60 min, washed, and mounted with ProLong™ Glass Antifade Mountant (P36982, Thermo Fisher Scientific).

### Fixed-cell imaging

Cells were seeded on coverslips (13 mm No.1S, MATSUNAMI) the day before fixation. Confocal microscopy was performed using a LSM880 with Airyscan (Zeiss) with a 63×1.4 Plan-Apochromat oil immersion lens or 100×1.46 alpha-Plan-Apochromat oil immersion lens. Images were analyzed and processed with Zeiss ZEN 2.3 SP1 FP3 (black, 64 bit) (ver. 14.0.21.201) and Fiji (ver. 2.0.0-rc-69/1.52p).

### Live-cell imaging

Live-cell imaging was performed using LSM880 with Airyscan (Zeiss) equipped with a 100×1.46 alpha-Plan-Apochromat oil immersion lens and Immersol^TM^ 518F/37°C (444970-9010-000, Zeiss). The day before imaging, cells were seeded on a glass bottom dish (627870, Greiner bio-one) with DMEM^gfp^-2 (MC102, evrogen) containing 10% FBS, PSG, and rutin (20 μg/mL) (30319-04, nacalai tesque). During live-cell imaging, the dish was mounted in a chamber (STXG-WSKMX-SET, TOKAI HIT) to maintain the incubation conditions at 37°C and 5% CO_2_. Images were acquired at intervals of 6 sec, analyzed and Airyscan processed with Zeiss ZEN 2.3 SP1 FP3 (black, 64 bit) (ver. 14.0.21.201) and Fiji (ver. 2.0.0-rc-69/1.52p).

### Quantification of the number of TBK1 puncta in the Golgi

Images of TBK1-mNeonGreen and mScarletI-GM130 were thresholded using Yen’s method with Fiji. TBK1- and GM130-positive regions of interest were extracted by multiplying the binarized TBK1 image by the binarized GM130 image. TBK1- and GM130-positive regions of interest were defined using the “Analyze Particles” command in Fiji on the binary thresholded image. The same processing was applied to the images of TBK1-mNeonGreen and mScarletI-Rab6.

### Quantification of the number of TBK1 puncta

Images of TBK1-mScarletI were thresholded using Yen’s method with Fiji. TBK1-positive regions of interest were defined using the “Analyze Particles” command in Fiji on the binary thresholded image.

### Statistical analysis

Statistical significance was determined with two-tailed Student’s t-test (Fig. 4C and Fig. 5F) or Tukey-Kramer’s test (Fig. 5C); NS, not significant (*P*>0.05). Data shown are representative of three independent experiments.

## Author contribution

H.K. designed and performed the experiments, analyzed the data, interpreted the results, and wrote the paper. Y.K. designed the experiments and interpreted the results. K.M. designed the experiments, analyzed the data, interpreted the results, and wrote the paper. T.T. designed the experiments, interpreted the results, and wrote the paper.

## Competing interests

The authors declare no competing interests.
