# Supplementary material for: Specific association of TBK1 with the trans-Golgi network following STING stimulation
Source: Cell Struct Funct. 2022 Feb 5;47(1):19–30. doi: 10.1247/csf.21080 (PMC10511044; doi:10.1247/csf.21080)
Supplement: Supplementary file 1 — Fig. S1 [file csf_47_21080_1.pdf]

# Supplementary Figure 1

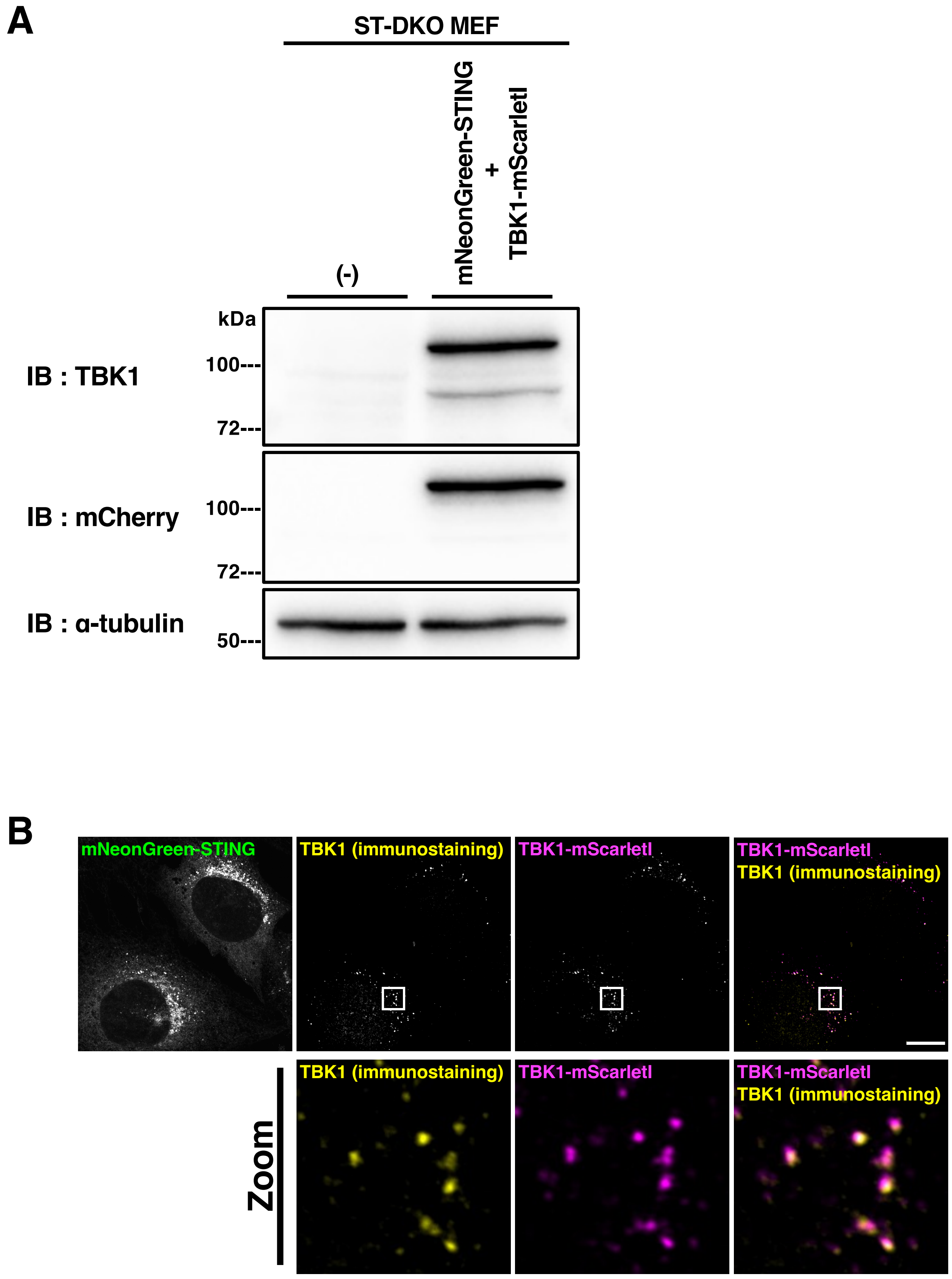


**Figure S1. Supplementary data related to Figure 1.**

(A) Cells were lysed and the cell lysates were analyzed by western blot with the indicated antibodies.

(B) ST-DKO MEFs reconstituted with mNeonGreen-STING and TBK1-mScarletI were stimulated with DMXAA (25 μg/mL) for 60 min. Cells were fixed, permeabilized, and stained for TBK1. Scale bars, 10 μm.
