# Supplementary material for: Specific association of TBK1 with the trans-Golgi network following STING stimulation
Source: Cell Struct Funct. 2022 Feb 5;47(1):19–30. doi: 10.1247/csf.21080 (PMC10511044; doi:10.1247/csf.21080)
Supplement: Supplementary file 2 — Fig. S2 [file csf_47_21080_2.pdf]

Supplementary Figure 2

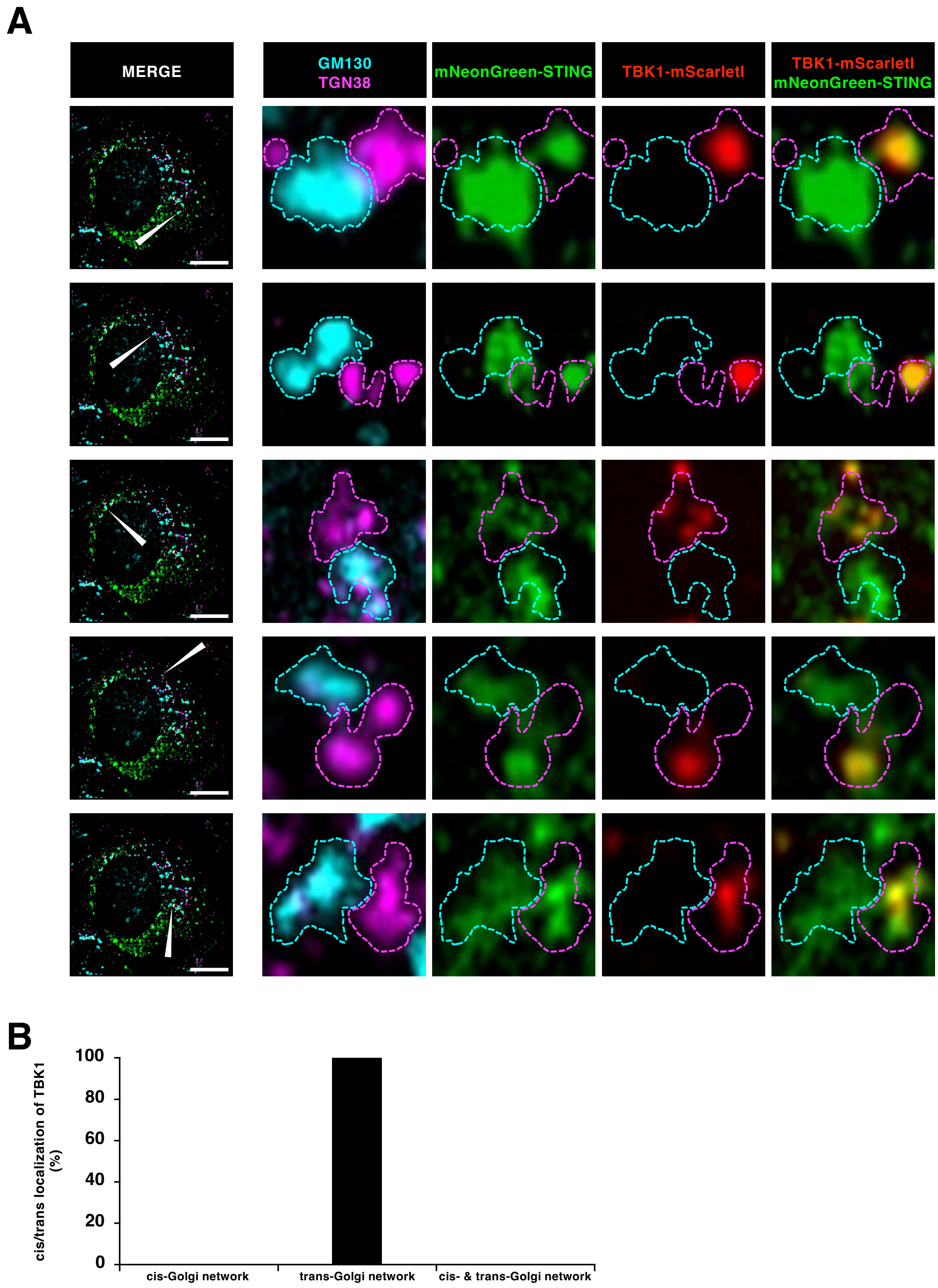

Figure S2. Representative images related to Figure 2

(**A**) Mini-Golgis indicated by arrowheads in the cell images at the left column were magnified. The *cis*- and *trans*-regions of the mini-Golgi were outlined. Scale bars, 10 µm.

(B) Quantification of TBK1 localization in the mini-Golgi. Thirty mini-Golgis from 5 cells were examined.
