# Supplementary material for: Specific association of TBK1 with the trans-Golgi network following STING stimulation
Source: Cell Struct Funct. 2022 Feb 5;47(1):19–30. doi: 10.1247/csf.21080 (PMC10511044; doi:10.1247/csf.21080)
Supplement: Supplementary file 3 — Fig. S3 [file csf_47_21080_3.pdf]

Supplementary Figure 3

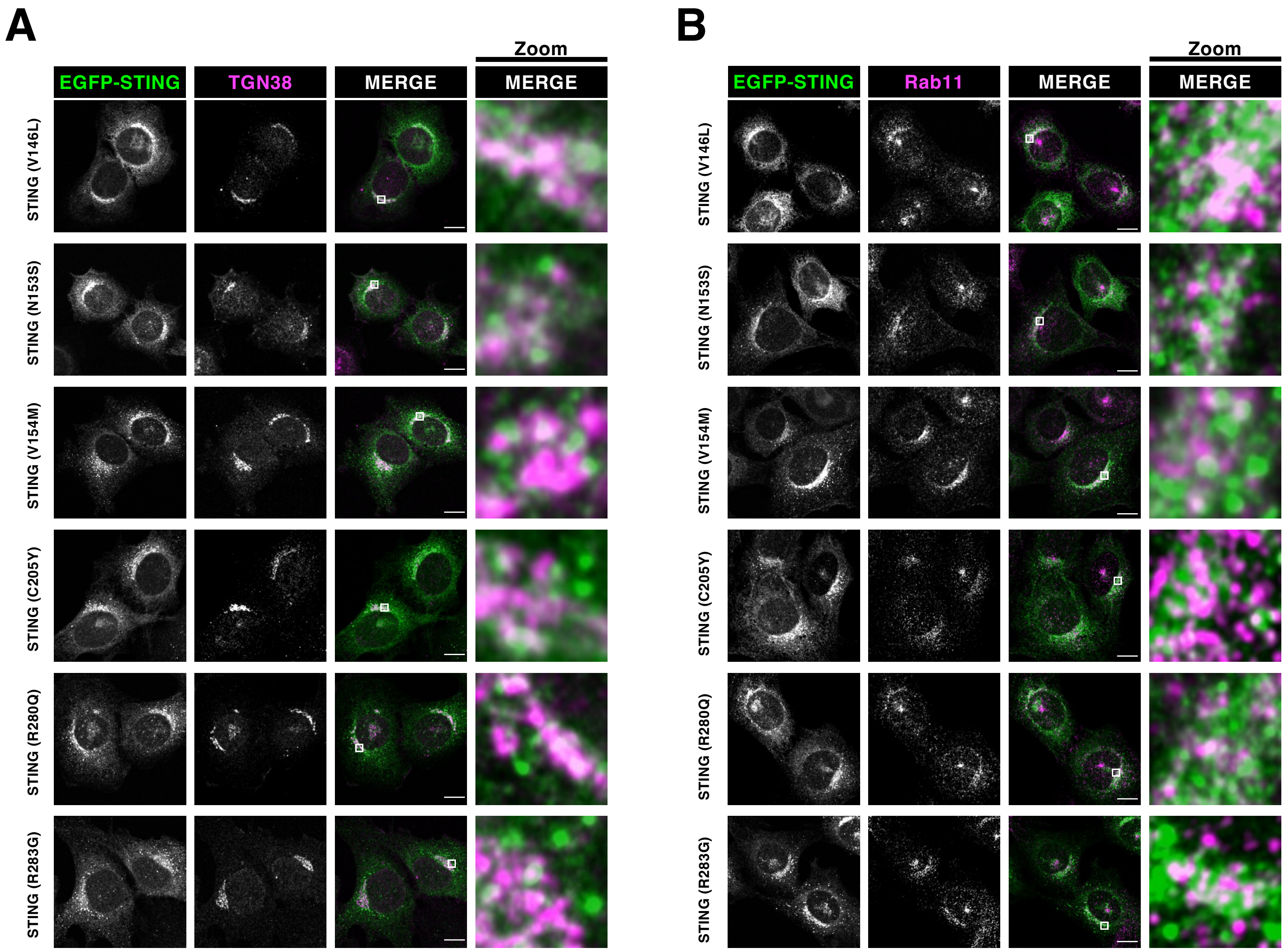

Figure S3. At steady state, the SAVI variants localize at the Golgi and recycling endosomes.

(A) ST-DKO MEFs reconstituted with EGFP-STING (SAVI) and TBK1-mScarletI were fixed, permeabilized, and stained for TGN38. Scale bars, 10 μm.

(B) ST-DKO MEFs reconstituted with EGFP-STING (SAVI) and TBK1-mScarletI were fixed, permeabilized, and stained for Rab11. Scale bars, 10 μm.
