# Supplementary material for: Specific association of TBK1 with the trans-Golgi network following STING stimulation
Source: Cell Struct Funct. 2022 Feb 5;47(1):19–30. doi: 10.1247/csf.21080 (PMC10511044; doi:10.1247/csf.21080)
Supplement: Supplementary file 4 — Fig. S4 [file csf_47_21080_4.pdf]

Supplementary Figure 4

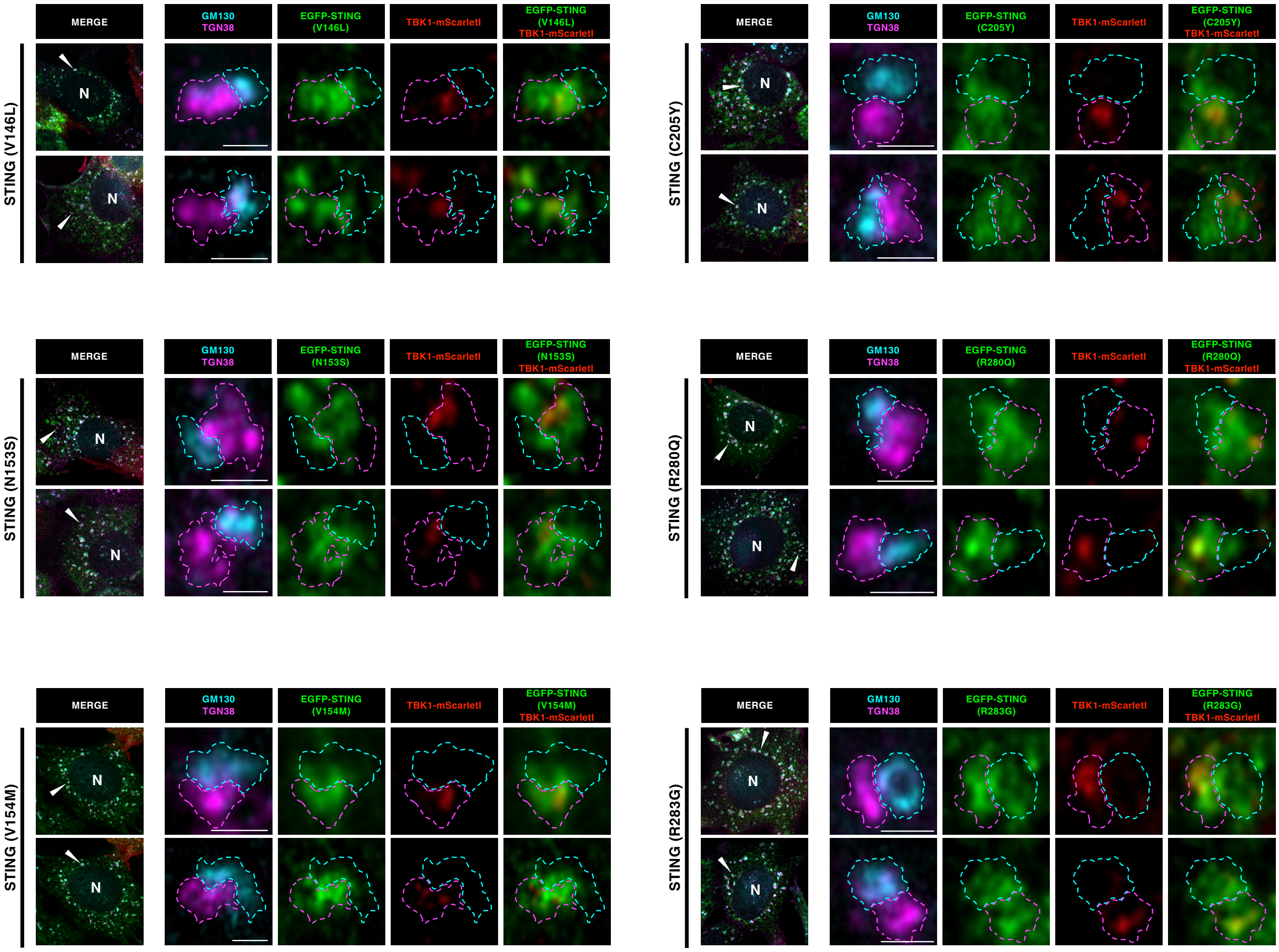

Figure S4. Representative images related to Figure 3

Mini-Golgis indicated by arrowheads in the cell images at the left column were magnified. The *cis*- and *trans*-regions of the mini-Golgi were outlined. Scale bars, 1 µm.
