# Supplementary material for: Specific association of TBK1 with the trans-Golgi network following STING stimulation
Source: Cell Struct Funct. 2022 Feb 5;47(1):19–30. doi: 10.1247/csf.21080 (PMC10511044; doi:10.1247/csf.21080)
Supplement: Supplementary file 5 — Fig. S5 [file csf_47_21080_5.pdf]

Supplementary Figure 5

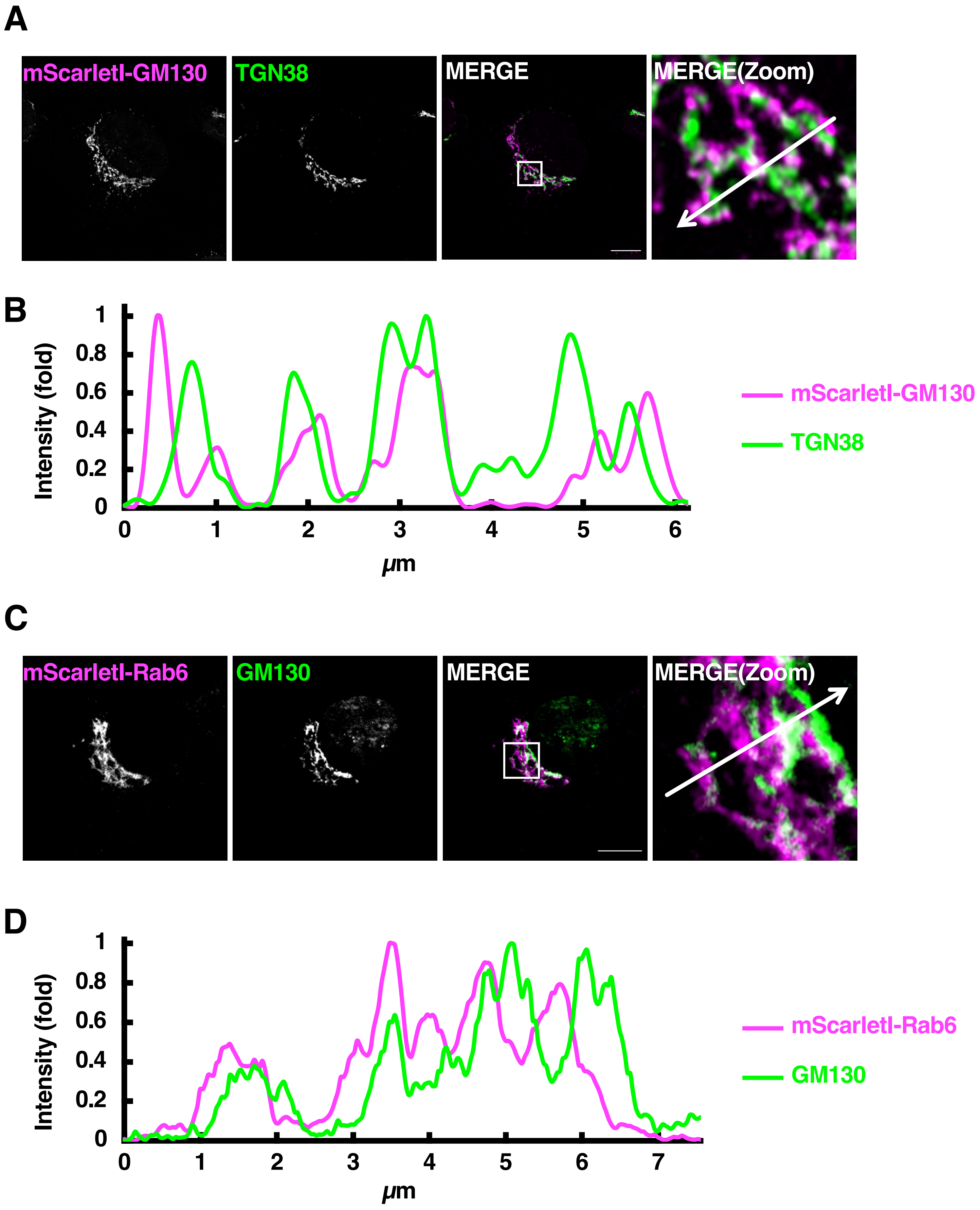

Figure S5. Co-localization analysis of CGN and TGN proteins with Airyscan super-resolution microscopy

(**A**) mScarletI-GM130 expressing MEFs were fixed, permeabilized, and stained for endogenous TGN38 (a TGN protein). (**B**) Fluorescence intensity profile along the arrow in (A) is shown. (**C**) mScarletI-Rab6 expressing MEFs were fixed, permeabilized, and stained for endogenous GM130 (a CGN protein). (**D**) Fluorescence intensity profile along the arrow in (C) is shown. Scale bar, 10 µm.
