# Supplementary material for: Specific association of TBK1 with the trans-Golgi network following STING stimulation
Source: Cell Struct Funct. 2022 Feb 5;47(1):19–30. doi: 10.1247/csf.21080 (PMC10511044; doi:10.1247/csf.21080)
Supplement: Supplementary file 6 — Fig. S6 [file csf_47_21080_6.pdf]

Supplementary Figure 6

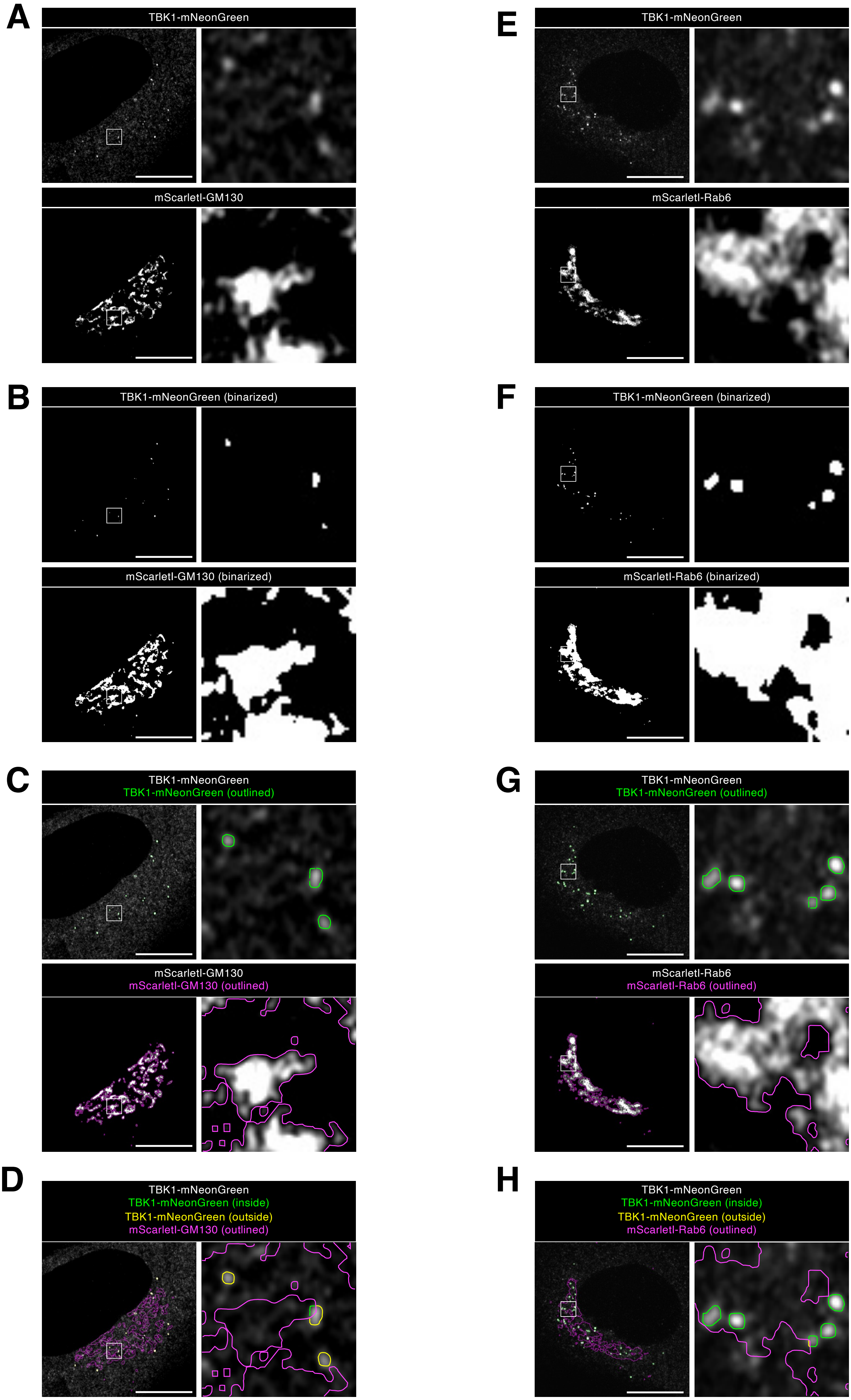

Figure S6. TBK1 translocates from the cytosol to TGN in living cells

The images after 21-min stimulation in Figure 4 were analyzed. Scale bar, 10 μm.

(**A**) The fluorescence images of TBK1 and GM130.

(**B**) Binarized images of (A).

(**C**) Outlines of the binarized images of (B) were obtained and merged with the images of (A).

(**D**) Outlines of TBK1 (green or yellow) and GM130 (magenta) were merged with fluorescence images of TBK1 (grey). TBK1 puncta inside or outside CGN were outlined with green or yellow, respectively.

(**E**) The fluorescence images of TBK1 and Rab6.

(**F**) Binarized images of (E).

(**G**) Outlines of the binarized images of (F) were obtained and merged with the images of (E).

(**H**) Outlines of TBK1 (green or yellow) and Rab6 (magenta) were merged with fluorescence images of TBK1 (grey). TBK1 puncta inside or outside TGN were outlined with green or yellow, respectively.
